# A qualitative investigation of optimal perinatal health: the perspectives of south Asian grandmothers living in southern Ontario, Canada

**DOI:** 10.1186/s12884-020-2762-0

**Published:** 2020-02-17

**Authors:** Sujane Kandasamy, Rebecca Anglin, Leila Gaind, Dipika Desai, Gita Wahi, Milan Gupta, Sonia S. Anand

**Affiliations:** 1 Department of Health Research Methods, Evidence & Impact, McMaster University, Hamilton, Canada; 2 Department of Psychiatry & Behavioural Neurosciences, McMaster University, Hamilton, Canada; 3 Department of Medicine, McMaster University, Hamilton, Canada; 4 The University of Notre Dame Australia, Fremantle, Australia; 5 Population Health Research Institute, Hamilton Health Sciences, Hamilton, Canada; 6 Department of Pediatrics, McMaster University, Hamilton, Canada; 7 Department of Medicine, University of Toronto, Toronto, Canada; 8 Li Ka Shing Knowledge Institute, St. Michael’s Hospital, Toronto, Canada

**Keywords:** Perinatal health, Pregnancy, Minority heath, South Asian, Grounded theory

## Abstract

**Background:**

Perinatal health-seeking behaviours are influenced by various factors, including personal beliefs. South Asian women, who often live within a wide kinship system, can be influenced by the advice and guidance of their mothers and/or mothers-in-law.

**Methods:**

To explore the cultural health perceptions of South Asian grandmothers within this context, we used constructivist grounded theory to sample and interview 17 South Asian grandmothers who reside in Southern Ontario, Canada. Interviews were audio-recorded, transcribed verbatim, and coded/analyzed by three independent coders.

**Results:**

Many grandmothers emphasized that the preconception phase should focus on building healthy habits around nutrition, physical activity, and mental wellness; the pregnancy period should encompass an enriched environment (positive relationships, healthy routines, nutritional enhancement); and the postpartum phase should emphasize healing and restoration for both the mother and newborn (self-care, bonding, rebuilding healthy habits). Many of the grandmothers conceptualized these stages as a cyclical relationship where healing and restoration transitions gradually to re-establishing healthy habits before having a subsequent child. They also expressed responsibility in supporting their daughters and/or daughters-in-law with their family units and encouraging the transfer of perinatal health information.

**Conclusions:**

South Asian grandmothers are involved in supporting the family units of their children and involving them in perinatal health programming can be an effective way to translate health knowledge to South Asian women.

Video abstract. In order to impact a broad, diverse audience of community members, we collaborated with a South Asian film-maker to distil the research findings, write an impactful script, and produce a short digital story based on the research findings. Currently available on social media (https://www.youtube.com/watch?v=tjcNUVOwatU), the film was celebrated with a CIHR Institute for Human Development, Child and Youth Health Video Talks Prize in 2016.

## Introduction

Maternal health-seeking behaviours of South Asian women can be impacted by their mothers, grandmothers, and other external family members. To supplement the quantitative data from our South Asian Birth Cohort (START), we aimed to investigate the specific role of grandmothers and their health perspectives of perinatal health using a community-focused qualitative study. This paper provides some background about the factors that influence maternal health behaviours, a profile of Canadian South Asians, the methods used to conduct this study, the results of the qualitative analysis, and a discussion of how the findings connect to the broader literature on how grandmothers advice impact the prenatal and postnatal behaviours of women. In our findings section, we aim to discuss how grandmothers perceive their role within their families and the way they understand and pass on prenatal and postnatal health advice to their daughters, daughters-in-law, and granddaughters. These ideas were then conceptualized into a schematic and developed into a digital story (visual abstract).

## Background

South Asians—people who were born in or can trace their ancestry to India, Pakistan, Bangladesh, Sri Lanka, Nepal—suffer high rates of type 2 diabetes and cardiovascular disease [1]. The metabolic risk factors for these conditions are influenced by the intrauterine environment, genetic factors, and maternal and paternal behaviors.

The maternal health-seeking behaviours of South Asian women can be influenced by their wide kinship networks, particularly by the “gatekeeper” role played by senior women (such as grandmothers). In a wide array of non-Western cultural contexts, grandmothers have been recognized for the critical role they play in advising and influencing younger generations [2–11]. Seen as figures of maternal wisdom and expertise, grandmothers have been identified as “learning institution [s],” responsible for transmitting their knowledge and prowess to expectant mothers both within the immediate family and the greater community [3].

Prior studies on grandmothers’ advice have focused on decisions about breastfeeding and infant nutrition, showing that the maternal grandmother exerts the most influence on feeding practices [12, 13]. Evidence also shows that South Asian mothers are more likely than their white Caucasian counterparts to receive advice about their pregnancy and breast feeding from other family members including grandmothers [14]. A single study of South Asian grandmothers’ influence on breast feeding in England found higher rates of breast feeding among a sample of women where the grandmothers were well-informed about the benefits of breastfeeding [14]. As emerging evidence demonstrates the importance of maternal behaviours on longitudinal health-outcomes, it becomes critical to identify the different social, cultural, and environmental factors that influence expecting mothers. To our knowledge, besides studies on infant nutrition and breastfeeding, there are no studies assessing South Asian grandmothers’ advice or beliefs about optimal health behaviours during the perinatal period.

### Demographic and health profile of Canadian SOUTH ASIANS

Representing over 4% of the total Canadian population, South Asians compose the largest subset of non-European ethnic groups in Canada [15]. Over 68% of Canadian South Asians were born outside of Canada with the majority having immigrated within the last decade and residing in the provinces of Ontario (62%) and British Columbia (22%) [15]. Seniors of South Asian origin are more likely than seniors of other ethnicities to live with family members and relatives, oftentimes supporting these units with domestic care and child rearing practices [16].

Evidence supports that the high rates of type 2 diabetes and cardiovascular disease faced by South Asians can be influenced by metabolic factors “programmed” in the developing fetus [17]. Comparative birth cohort studies from India, UK, and Canada have shown that although South Asian mothers are younger and have a lower body mass index (BMI) compared to European counterparts, South Asian babies are lower weight yet have comparable subscapular skin fold thickness, suggesting a greater adipose tissue percentage or fatness [18–20]. In addition, these South Asian offspring are observed to have increased adiposity, glucose, insulin, and leptin concentrations later in life [18]. It is possible that differences in maternal characteristics, gestational age, and pregnancy factors (i.e. maternal nutrition, gestational diabetes, hypertension, health beliefs) may explain these differences. To investigate the factors that predispose South Asian peoples to type two diabetes and other cardio-metabolic conditions, we have established the prospective South Asian Birth Cohort study in Canada (START) [21] in which more than 1000 mother-infant dyads have been enrolled. To supplement the quantitative information that we generate from the START study, we aimed to also collect a rich source of qualitative data in order to gain a more holistic perspective on the sociocultural factors that may influence expecting mothers within South Asian communities in Canada. Thus, the objective of this study was to use a constructivist grounded theory approach to explore the perinatal health beliefs of South Asian grandmothers living in Canada (not limited to START grandmothers). Furthermore, we aimed to enhance the dissemination of this study through the development of a culturally-meaningful integrated knowledge translation project (digital story) that can be used to connect with community members and share the results of this study.

## Methods

Individual qualitative interviews were conducted with grandmothers of South Asian origin, ensuring that their beliefs were captured using an ethnically-sensitive lens. This was made certain by upholding a non-threatening environment (interview location was decided by the participant and often took place in their home), conducting interviews in the participant’s preferred language (see Table 1 for a breakdown of the various South Asian languages used in this study), and upholding judgement-free interactions with research participants (acknowledging culturally-specific phenomena and beliefs regarding optimal maternal health behaviours through the interview design and delivery).

**Table 1:** Demographic details of study participants (*n* = 17)

Total Number of Grandmothers Interviewed	17
Median Number of Grandchildren Each	3
Country of Origin	
India	8 (47%)
Pakistan	4 (24%)
Sri Lanka	5 (29%)
Median Years Living in Canada	25
Mother Tongue	
Punjabi	7 (41%)
Hindi	2 (12%)
Tamil	5 (29%)
Gujarati	1 (6%)
English	2 (12%)
Religion	
Hindu	6 (35%)
Sikh	6 (35%)
Zoroastrian	3 (18%)
Unknown	2 (12%)
Highest Level of Education	
Less than High school	4 (23%)
High school	2 (12%)
College diploma or equivalent	3 (18%)
Bachelor’s degree	3 (18%)
Professional degree (eg: MD, DC)	1 (6%)
Unknown	4 (23%)

Guided by a semi-structured interview guide (Additional file 1), the questions posed by the interviewers touched on precise topics, yet also facilitated an open discussion. This was achieved by using flexible probes such as “What do you think [about any given topic]?” Data collection and data analysis were dynamic and iterative and subsequent interviews followed-up or inquired in more detail about emerging codes or themes. Data analysis consisted of line-by-line initial coding, focused coding, and thematic analysis, as per a constructivist grounded theory approach [22]. All data analysis was completed manually and independently in triplicate. The three coders (RA, SK, and LG) coded the data using a staged approach and held several face-to-face meetings to discuss the data. NVIVO-9 software was used to organize the coding.

### Study sample and recruitment

17 South Asian grandmothers from the Peel and York regions of Southern Ontario participated in the study. Participants originated from India, Pakistan, and Sri Lanka and spoke one of the following languages: Punjabi, Hindi, Tamil, Gujarati, or English. 53% of the interviews were conducted in a native language other than English. See Table 1 for additional demographic characteristics of the participants.

Grandmothers of South Asian origin living in Canada were recruited through community advertisements and recruitment events. In addition, participants in the START study were asked if their mothers or individuals who play a motherly role in their life would be willing to participate. These individuals were then contacted by phone and provided information about the study and invited to attend an interview in the language of their choice. Once the grandmothers were recruited into the study and provided written informed consent, a member of the research team scheduled the interview at a convenient time for the participant at one of our START recruiting centres, centrally located in Brampton, Ontario at the Canadian Cardiovascular Research Network Office. If participants could not travel to the centre, the interview was conducted in their home without any other members of the family present in the room to ensure confidentiality. All interviews were conducted in the participants’ native language if they were not fluent in English. All participants currently reside in Canada and have children who also live in Canada.

Participants were sampled using a non-probabilistic method until data saturation was achieved, with a total of 17 grandmothers participating in this study. The saturation point was determined by consensus from the research team and was defined as the point where additional data collection did not lead to new data or insight. The iterative methodology enforced a constant review of key informants that would help solidify the themes. These participants were identified through community partnerships in Peel and York regions and through the guidance of previous interviewees. For example, to investigate if any differences existed between the beliefs of maternal and paternal grandmothers, early participants (who were maternal grandmothers) consulted their personal social networks to guide us to participants that were paternal grandmothers.

### Inclusion criteria

1. Participants were grandmothers of South Asian ethnicity (defined as originating from India, Pakistan, Bangladesh, Guyana, Trinidad, Nepal or Sri Lanka). ‘Grandmother’ was defined as having a pregnant daughter or daughter-in-law or grandchildren by their daughter or son (adopted or biological)
2. Participants had to agree to the oral interview and possible follow-up

### Data collection

A semi-structured interview guide was used to navigate the qualitative interviews (Additional file 1). This enabled the interviewee to freely discuss their beliefs while still allowing the interviewer to ask for further explanations when needed. An example of a question includes “What do you think are important things for a mother-to-be to do when she is trying to get pregnant to make sure she and the baby are healthy?”

All interviews were audio-recorded to facilitate transcription and translation, which was required for analysis. Each interview lasted approximately 1 h in length. The interview questions were designed specifically to elicit the grandmother’s beliefs regarding optimal health behaviours for a woman (1) before pregnancy, (2) during pregnancy, (3) the first 6 weeks postpartum, and (4) optimal behaviours for the family with the new baby in the first year of life. Although we loosely inquired about diet, sleep, activity levels, social support, mental health, and intimate partner relationships, the interview questions supported an environment where the participants could focus on the health factors that they felt were most conducive to optimal health. Participants were debriefed and provided with the opportunity to ask questions after the interview was completed.

### Data analysis

All interviews were conducted by three trained interviewers who were fluent in either Punjabi, Hindi, Tamil, Gujarati, and/or English. All interviews were audio recorded and transcribed verbatim. Those that were not conducted in English were transcribed in the native language first and then translated. If there was an ambiguity in translating particular words, this was highlighted and the correct translation was confirmed with the interviewer and where necessary, with the participant. Multiple layers of coding were performed (initial and focused coding) using a staged approach. Memo-writing occurred after each interview, allowing the researchers to take note of emerging themes on an on-going basis. The coded data was reviewed and a constant comparison technique was used to identify emergent themes, concepts and linkages [22, 23]. The data was closely adhered to with sensitivity to emerging subthemes and when saturation was reached, no further interviews were held. After identification of themes, participants were invited to have a final interview where the themes were presented back to them for member-checking. Throughout, analytic memos and field notes were kept to maintain reflexivity and self-awareness. For example, field notes included a reflection of how the overall interview went, what the interviewer’s role was perceived to be, and any other observations of the interactions between the researcher and the participant. These reflections were reviewed with the research team after each interview and modifications were made to future interviews to minimize personal and intellectual biases.

## Results

### The role of South ASIAN grandmothers

Many grandmothers believed they play an important role in the familial hierarchy and saw themselves as a figure of wisdom and experience, often advising their daughters and/or daughters-in-law on matters regarding food, activity, and optimal maternal behaviours. Many also said their role was to help preserve the cultural values and traditions that have been upheld by previous generations. For example, one grandmother responded with *“we keep our culture and religion (Participant 013)”* when asked about the role she played within her family. Another grandmother said she takes responsibility in “*teaching about culture and cooking”* (Participant 007).

Furthermore, many grandmothers felt they maintain a very positive relationship with their grandchildren and enjoy taking an active role in their health and well-being. This relationship was associated with a great sense of fulfillment and purpose. For example one grandmother said, *“I am always interested in my grandchildren’s growth, studies, and health. It’s my choice too. I love to take care of them (Participant 003).”* Another grandmother said, *“we have such an active role in their [our grandchildren’s] upbringing (Participant 002).”* In some cases, taking an active role in the lives of their grandchildren result in them “worrying about whether they will do well” (Participant 001).

### Attitudes toward advice-giving

Generally, the grandmothers’ attitudes towards giving advice can be categorized in three ways and may change depending on the situation: **a) Enforcing:** this style of giving advice is steadfast with the belief that it will be followed. Stemming from the perceived role of the grandmother as a figure of wisdom and experience, one who is responsible for taking care of the family through her actions and the advice she provides. For example, one grandmother said *“We advise them on each matter and they do follow them (Participant 012)”*; b) **Intervenes when necessary:** this style of giving advice is described as intervening only when the right opportunity arises. This stems from the belief that although the new mother will act in the best interest of the child, she may need guidance around certain behaviours. For example, one grandmother said, *“That should be her choice as to what she wants to eat or drink, but as to what you want to advocate it’s different … Like iron … she should take supplements if she’s low on iron (Participant 004)”*; **c) Imparts when asked**: This style is described as giving advice only when requested and not necessarily advising otherwise. Grandmothers who provide advice this way oftentimes acknowledge generational and intercultural differences in terms of child-rearing. For example, one grandmother said, *“I normally don’t give advice because I don’t like to impose myself … But yes whenever she does need any advice I do want to share my advice and she follows it. At other times I just give a suggestion for her to weigh the pros and cons and then decide for herself (Participant 006).”*

### The health beliefs of grandmothers

Our data reveals that South Asian grandmothers garner their health perceptions from three main sources of knowledge: experiential (personal experience), medical-based, and community-based/cultural. Many also identified a distinction between what behaviours are considered optimal during the three main stages of the perinatal period. The pre-conception period was characterized to be reserved for healthy habit building; the pregnancy period was considered to be a time where the mother surrounds her mind and body in an enriched environment; the postnatal period was thought to include behaviours that support healing and restoration for both the mother and the newborn.

#### The preconception phase

To ensure optimal health, many grandmothers recommended that their daughters or daughters-in-law practice building healthy habits during the pre-conception period. With a special emphasis on mental health, nutrition, and physical activity, grandmothers believed that women should engage in stress reduction practices (e.g. yoga, meditation) and occupy themselves in a positive mental and physical state. For example, one grandmother said, *“your mind should be healthy. You have to keep your mind peaceful (Participant 005).”*

Grandmothers believed that keeping one’s mind at peace is an important step in taking care of others (including future children). Many emphasized the importance of moderation in one’s diet, implying that eating well is characterized by balance, instead of specific or extreme food restrictions. For example, one grandmother explained that it is important to have *“moderation in everything. I don’t believe in extremes (Participant 008).”* Another grandmother said, *“It should be a well-balanced diet, not something in particular (Participant 009).”* Many felt that during the pre-conception phase, eating all foods in moderation was important to building healthy habits.

Overall, the pre-conception period was considered to be a time where the body is being prepared for supporting the optimal growth of a baby. As a result, women should build health habits that are conducive to ensuring metal wellbeing, nutritious eating, and non-strenuous physical activity.

#### The pregnancy phase

Many grandmothers believed that in order to support the growth and delivery of a healthy baby, the pregnant woman has to reflect and surround herself in an ideal environment. This is best achieved by continuing to practice the healthy habits that were built during the pre-conception stage. An “Enriched Environment” was upheld by keeping positive relationships and support systems, maintaining healthy routines, nutritional enhancement, and moderate, non-strenuous exercise. The main reason that many grandmothers believed that an enriched environment was the key to optimal health during pregnancy was because of the belief that everything the pregnant woman surrounded herself with would be imprinted onto the baby growing inside of her, including thoughts and conversations that she may have. For example, two different grandmothers provided the following explanations:

*“After the first three months I think the baby starts to hear what is going on in the family so it’s good to have good thoughts, read good books and listen to good music, and no fighting, you know the baby can tell when the mother’s upset. So psychologically try to have happy thoughts (Participant 004).”*

*“Mentally soothe yourself you know, listen to calming music. You know all of that is beneficial to the baby and I guess it’s all tied into the same theme. The mother is calm the baby is going to be calm. The mother eats right the child will eat right so it’s all like a tree giving forth good food (Participant 005).”*

Maintaining an enriched environment was believed to primarily follow from the preservation of healthy habits. In addition to keeping one’s mind at peace, some grandmothers indicated that this period should include surrounding oneself with religious and intellectual stimuli like books and pictures, and also engaging in prayers and ceremonies. These activities are seen as important not only for the expecting mother’s own mental health, but for the well-being of the child. For example, one grandmother explained this: *“Read good books, religious books … Read good novels and watch religious movies, or do prayer. Meditation (Participant 003).”* Another participant said, *“You can read books, visit spiritual institutions, listen to songs, radio, read newspapers, and talk to people (Participant 007).”* Many grandmothers also spoke of the mother-child connection that exists, and the ways in which the mother’s own health and state of mind can influence the child in-utero. For this reason, maintaining a positive state of mind was seen as imperative to the health of the child, and many grandmothers spoke of different ways for the expecting mother to remain happy and stress-free. This often included being social, interacting with friends and family, and maintaining a sense of “normal” life and routine. Some grandmothers spoke of their own experience dealing with the stresses of pregnancy, and the importance of reaching out to support networks and asking for help when necessary.

There was an emphasis on nutritional enhancement in regards to adding special foods to the diet and replacing or replenishing vitamins. Many grandmothers believed that the pregnant woman eating particular foods would foster particular traits in the child in addition to informing his or her overall health and wellbeing. Smoking, drinking, and the use of drugs were generally seen as negative, both culturally and also due to the adverse health effects these substances can have. This viewpoint was generally extended to prescription medications during pregnancy, unless it is a last resort.

Consistent with the pre-conception period, light exercise was seen as important, especially in the management of excess weight gain. While food is seen as nourishing and healthy, there was still an understanding that the pregnancy period should not be one of gluttony and inactivity, and the mother must still remain healthy and active for herself and the baby. For example, two different grandmothers said, *“she should not gain too much of weight. Staying healthy and active is important (Participant 016)”* and that *“she should exercise and do household work. Sleeping entire day is not good, walking is a good exercise (Participant 017).”* Few grandmothers mentioned intentional, strenuous exercises such as weight bearing, running, or gym-related activities, and instead focused on walking. Some grandmothers also expressed concern regarding more rigorous activities, as they believe them to adversely affect the expecting mother’s health by increasing the risks of abortion. For example, one grandmother said she would recommend the following: *“Walking. I wouldn’t do anything too strenuous in excess you know, no jogging or anything just walking on a treadmill or something (Participant 014).”*

#### Post-partum phase

The postnatal period was described to be a time of healing and restoration for the mother and the baby. First and foremost, many grandmothers emphasized the importance of self-care during this period, ensuring that the mother regains both the physical and mental strength necessary to take care of the baby. This included activities such as getting adequate rest, eating well, taking soothing baths, listening to calming music, and maintaining a positive mental state. For example, one grandmother said, *“she should look into her own self first and see what used to make her happy, what used to give her more satisfaction … Maybe read, hear good music, go out … Essentially you should love your own self and your own company. If you can manage to get that down you will never be sad (Participant 005).”*

Many grandmothers mentioned that friends and family can play an important role during this period by offering their love and support for the mother and new baby. Raising the child was seen as a collective responsibility, and many grandmothers assumed an active role in this regard. Furthermore, the ongoing practical and emotional support was seen as imperative to the mother’s mental health, helping her during this period of stress and change. For example, one grandmother said, *“I think that the mother needs somebody there every day for six weeks. My daughter is still taking antidepressants ‘til now, from the first baby … I don’t know how much care she had because you can’t interfere with certain things, and I don’t think she had the support like we had back home in our country, and I think she suffered because of that (Participant 008).”*

Following this initial period of recovery and restoration, many grandmothers encouraged their daughters and daughters-in-law to start re-building healthy habits and re-integrating into the flow of normal life (re-build her routines). Many felt this included behaviours such as learning to maintain a work-home balance, eating healthy, and upholding a good social network. For example, one grandmother explained that, *“in the first year she should gradually get back into her own routine life. The longer she waits, the harder it becomes (Participant 012).”*

Furthermore, grandmothers expressed that the mother bonds with the child through co-sleeping, massages, stretching, and breastfeeding. For example one grandmother explained the importance of helping the baby stretch, *“you cross their arms, you cross their legs, you have their legs touching their head, you know so it’s a stretching thing and the baby’s so relaxed they go to sleep (Participant 014).”* Another grandmother said, *“even now my daughter-in-law massages the back and the tummy, soothes, reads to them, lullabies (Participant 009).”*

Breastfeeding was seen as important and often linked to reduced infection and sickness in the baby. It was generally believed that a child who was breastfed will be healthier. For example, one grandmother said, *“for one thing, breastfeeding helps the baby have a healthy growth. Secondly it sort of builds a bond with the mother and the child. I know there’s a natural bond that comes with the mother and the child and a child gets more immunity. A [breastfed] child is healthier (Participant 010).”*

Similar to the notion of imprinting, many believed that the baby can sense when the mother is anxious or sad, and therefore her mental state can have a profound effect on the child. Many grandmothers explained that the “mother-child connection” is imperative to avoid any stress or fighting that could influence the child’s temperament or impede the mother’s ability to take good care of the child. As opposed to food being almost exclusively seen as nourishing during pregnancy, there was a focus on avoiding certain foods that would cause distress in the baby through breastfeeding (referred to as *Kirandhi* foods), as well as ensuring an adequate diet pattern full of healthy, fresh foods.

**Fig. 1:**
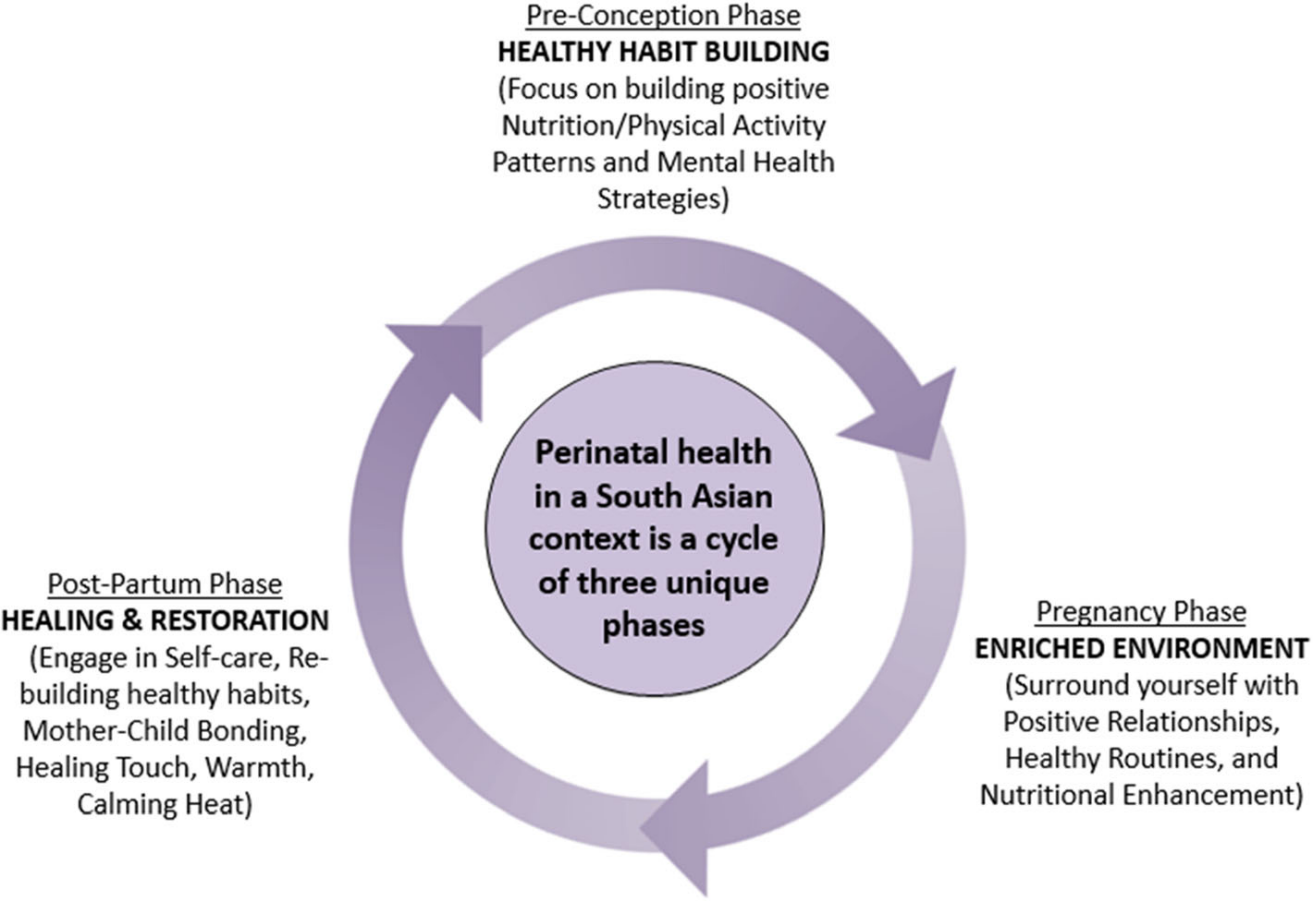
This framework illustrates the perinatal health perspectives of South Asian grandmothers. The grandmothers believe the preconception phase should include behaviours that promote building healthy habits; the pregnancy phase should include behaviours that enrich the surrounding environment of the mother and child. After the child is born, grandmothers recommend that women should focus their behaviours around healing and restoration, including re-building healthy habits. Grandmothers also suggested that a cyclical relationship should exist between these phases, as many South Asian women have more than one child

Overall, the grandmothers believed that the preconception phase begins far before planning to have a child and includes behaviours that promote building healthy habits (particularly around nutrition, physical activity, and mental wellness). They believed the pregnancy phase should include behaviours that enrich the surrounding environment of the mother and child. After the child is born, the woman should focus her behviours around healing and restoration, including re-building healthy habits. It is important that a cyclical relationship exists between these phases, as many South Asian women have more than one child (See Fig. 1). See Additional file 2 for additional supporting raw data for each theme.

## Discussion

This constructivist grounded theory approach to understanding the perinatal health beliefs of older South Asian women contributes to the discussion of the factors that influence the maternal health-seeking behaviours of South Asian women living in Canada. We have identified that older women play important roles within the family structure and may influence pregnant women’s actions through their understanding of pre and postnatal health. Participants identified that the preconception phase includes behaviours that promote building healthy habits. This phase transitions into pregnancy, which includes behaviours that enrich the surrounding environment of the mother and child (positive relationships, healthy routines, and nutritional enhancement). After the child is born, the woman should focus her behaviours around healing and restoration, including re-building healthy habits, which would take her into the enriched environment of a subsequent pregnancy.

As this study focuses on the advice that South Asian grandmothers give to expecting mothers, it is important to note the factors that inform their beliefs and the ways in which they deliver their advice. We identified three main sources of knowledge: experiential, medical-based, and community-based/cultural. Throughout the interviews, the grandmothers referred back to all three sources, in varying degrees, and with changing attitudes towards them. Although it varied from interview to interview and was context-dependent, there was a general consensus that the best advice was derived from one’s own experience, with additional support from a family physician or medical authority. Culturally specific sources of knowledge were still valued, but not necessarily above experience and Western medical advice. In general, the grandmothers were flexible in their beliefs, and provided advice that they felt was for the optimal benefit the child. This flexibility has applications to health programming because it facilitates various outlets and avenues for sharing knowledge with grandmothers—who, in their trusted roles within the family as those who provide guidance can be better supported. As South Asian grandmothers rely on medical information from physicians and healthcare providers, it is important that evidence-based perinatal health information is translated in a culturally-meaningful and timely fashion. For example, peer-based programs and family-centered clinic appointments can help keep South Asian grandmothers actively and productively involved in the care of their families.

Most grandmothers also acknowledged the propensity of South Asians to be at risk for heart disease and diabetes and when asked for probable causes, they cited both lack of exercise and poor diet. They were concerned about the abundant food choices and physical activity constraints that are the results of immigrating to a country of cold climate. For example, one grandmother said, “*Heart disease and diabetes. When they migrate and come into Canada they see a lot of foods that are so attractive that they may not have had back home so they tend to have a lot of that and over intake of the wrong type of foods gets them into these health issues (Participant 004).”* As many grandmothers acknowledged the challenges around eating healthy and engaging in adequate physical activity, we believe that extra supports need to be offered around building healthy habits during the pre-conception phase and re-building healthy habits during the postnatal phase. This could, for example, include involving elders in the design of physical activity programs (e.g. community family walks) so that they can help advocate and encourage women in their families to participate.

Recent longitudinal birth cohorts have provided important information about what factors influence the health and well-being of children. However, in many cases, the advice resulting from the studies was the same as the advice many grandmothers give [24]. Yet, grandmothers have rarely been considered as important figures in child development programs and educational initiatives [3]. Existing literature has identified several research biases that may in part account for this deficit, such as: the belief that grandmothers do not in fact influence child-rearing practices, the notion that the advice that grandmothers give is often out-dated and contributes negatively to the child’s wellbeing, the stereotype of the elderly as being uneducated and illiterate, and the idea that due to their age, grandmothers are incapable of learning new things and adapting their long-held beliefs to more modern practices [3]. However, studies have shown that especially in non-Western cultures, involving grandmothers in educational programs and initiatives can help ensure their success because they capitalize on existing familial structures and respect cultural norms [3]. To reiterate, since grandmothers are so involved in perinatal care, they are important participants of public health programming. Targeting educational-exchanges between grandmothers and health professionals can help tailor information that will be most beneficial and impactful for South Asian woman and thus, South Asian families.

### Strengths and limitations

The strengths of this study include the community-level knowledge translation efforts (in the form of a locally-produced video abstract), the rigor and safeguards built into our constructivist grounded theory approach (e.g. including the detail/depth of line by line coding, having multiple coders, conducting member-check and follow-up interviews with 5 participants), and celebrating the diversity that exists within South Asian communities by purposefully sampling participants that represent this heterogeneity (e.g. across languages and religions). In addition, conducting comparative studies to START antenatal and postnatal data will help us direct future knowledge translation opportunities that can promote healthy postnatal behaviours and interject upon unhealthy behaviours. Interviewing mothers, fathers, and other family members can also provide additional insights into how we can support Canadian South Asian families create an optimal environment for their children.

## Conclusions

Overall, this study contributes to the limited literature that is currently available on older South Asian women’s cultural perceptions and beliefs around prenatal and postnatal health. By carefully assessing these health beliefs and understanding the cultural context of these perceptions, we now have a better understanding of how we can work collaboratively to improve public health programming directed toward pregnant South Asian women. This includes the incorporation of the cyclical relationship that emphasizes specific behaviours during the distinct perinatal stages to help guide and support maternal behaviours that are culturally viewed as optimal for the health of both the mother and the child. In addition, it also contributes to the evidence that supports the inclusion of older women in the perinatal decision-making process—something that can help improve the dialogue between grandmothers and pregnant women in a way that capitalizes on behaviours that improves the wellbeing of South Asian families living in Canada.

## Supplementary information

**Additional file 1.** (Semi-structured interview guide) This file includes the semi-structured interview guide and probing questions.

**Additional file 2.** (Supporting raw data) This file contains additional supporting raw data from each theme.

## Data Availability

The corresponding author can share interview transcripts, based on reasonable request.

## Abbreviations

BMI: Body Mass Index
START: South Asian Birth Cohort Study
