# Supplementary material for: A qualitative investigation of optimal perinatal health: the perspectives of south Asian grandmothers living in southern Ontario, Canada
Source: BMC Pregnancy Childbirth. 2020 Feb 17;20:113. doi: 10.1186/s12884-020-2762-0 (PMC7026998; doi:10.1186/s12884-020-2762-0)
Supplement: Supplementary file 2 — Additional file 1. (Semi-structured interview guide) This file includes the semi-structured interview guide and probing questions. [file 12884_2020_2762_MOESM2_ESM.doc]

**Grandmother’s Advice Study Interview Guide**

***Demographics***

Please ask participant to spell their first and last name and their contact information (if they have agreed to be contacted again at a later date to receive feedback on the study).

***Information about the Grandmother***

What is your ethnicity?

What is your mother tongue? What language do you feel most comfortable to communicate in?

Do you practice any religion? What is your religious background?

In what country were you born?

- If not Canada, how long have you been in Canada?
- If you are born in Canada, what generation are you (first, second, etc)?

Do you have any dependents at home?

- If so, how many?
- What generation are they relative to you (i.e. children/ parents)?

What is your highest level of education?

What is your current occupation (or past career if retired)? If immigrant, what was your occupation in your previous country of residence/origin

Do you have any grandchildren? IF START COHORT GRANDMOTHER: Who referred you to us? Does she have children?

How would you describe your relationship with your daughter/daughter-in-law/niece (Select appropriate term)?

- Specifically, how would you describe your role in your family?
- How do you think your daughter/daughter-in-law (select term) views you? Do you think she follows your advice?

Tell me about your experience of being a grandmother.

- What have been the good things?
- What have been the difficult things?
- Do you think it is different having a grand-son or a grand-daughter?

Where did you learn about the best health for the mother before, during and after pregnancy?

***Main Questions and Corresponding Probes***

What do you think are important things for a mother-to-be to do when she is trying to get pregnant to make sure she and the baby are healthy?

- Should a mother-to-be make any changes to what she eats before she gets pregnant?
  - How do you know if she is getting enough adequate nutrition? What if she isn’t?
  - What do you think about vitamin intake for mothers-to-be?
  - Any other special foods or drinks the mother should take while she is trying to conceive?

What do you think are important things for a mother to do while she is pregnant to make sure she and the baby are healthy?

What do you think are the biggest health concerns for women when they are pregnant?

- Do you think that South Asian women have any unique health problems when they are pregnant?

What foods do you think a mother should eat when she is pregnant?

- Are there any foods you think a mother-to-be should stay away from/avoid during her pregnancy?
- Are there any other changes a mother should make to her diet when she is pregnant?
- What do you think about a mother eating seafood when she is pregnant?

How much weight do you think a mother-to-be should gain during pregnancy?

How much sleep should a mother get when she is pregnant? When should she sleep?

Should a mother continue to work at her job while she is pregnant?

- How much is good for her and how much would be bad?
- What are your thoughts about a mother-to-be and exercise during pregnancy? What kind of exercise(s) should she do?

What do you think about smoking and pregnancy?

- What do you think can happen to the mother-to-be if she smokes during pregnancy?
- What do you think can happen to the baby/fetus?

What do you think about drinking alcohol during pregnancy?

- What do you think can happen to the mother if she drinks alcohol during pregnancy?
- What do you think can happen to the baby/fetus?

What do you think are important things for a mother to do in the first 6 weeks after giving birth to make sure she and the baby are healthy? In the first year?

What do you think are the biggest health problems for women after giving birth?

Are there any particular problems for South Asian women?

Should a mother make any changes in her diet in the first 6 weeks after giving birth? If so, what?

- What foods should she stay away from?
- Are there any changes she should make to her diet if she is breastfeeding?

Should a mother go back to work after having a baby? If so, when?

Do you think it is safe for a mother to exercise after she has given birth?

- If so, when do you think she should start exercising?
- How much exercise should she do?
- What kind of exercise(s) should she do?

What do you think are the biggest health problems for babies?

How much should a baby weigh when they are born?

- How much weight should they gain in the first month?
- In the first 6 months?
- Do you think weight gain should be different for boys and girls?

How should a mother clean her baby? How often should she clean her baby? Are there any consequences to keeping the baby to clean or too dirty?

What do you think about vaccinating babies?

What are your thoughts about a mother breastfeeding? Formula feeding?

- Should a mother breastfeed or formula feed their baby? Why?
- If both, why and in what combination?
- What do you think are the health benefits (or other benefits) to breastfeeding?

What do you think are the health benefits (or other benefits) to formula feeding?

What do you think is the ideal time that a mother should breastfeed for?

Are there any traditional foods a baby should receive?

How much should a mother feed their baby?

Should it be different for boys versus girls?

How can you tell (signs) if a baby is getting too little or too much food?

When do you think the baby should start eating solid foods?

What is the first food a baby eats? (i.e. Pablum?)

What should a mother do if a baby has colic?

How much should a baby sleep? (Prompt for in the 1^st^ month, at 6 months, 1 year)

When do you think the baby should start sleeping through the night?

Where should a baby sleep? (Prompt for in the 1^st^ month, at 6 months, 1 year)

How should a baby sleep (ie front, back side)

What should a mother do to help their baby sleep? Or –“How would you advise a mother to help get her baby to sleep”?

How much sleep should a mother try to get? (Prompt for in the 1^st^ month, at 6 months, 1 year). What advice would you give her to improve her sleep?

When should a mother start toilet-training her child?

How should toilet-training be done?

When should a child be completely toilet-trained?

What should a mother do if she is feeling sad all the time when she is pregnant? What about after having the baby?

- Who should she talk to?
- How should she seek help?
- Do you know of any remedies?
- What about seeing a doctor?
- What about medical treatments such as counseling or antidepressants?

What should a mother do if she is feeling anxious all the time when she is pregnant? What about after having the baby?

- Who should she talk to?
- How should she seek help?
- Do you know of any remedies?
- What about seeing a doctor?
- What about medical treatments such as counseling or antidepressants?

What impact do you think being sad and anxious all the time during pregnancy can have on the baby? What about after having the baby?

What do you think a mother-to-be should do if her husband yells at her when she is pregnant? What about after having the baby?

What do you think a mother-to-be should do if her husband acts violently with her while she is pregnant? What about after having the baby?

Do you talk to your family/friends about health in general?

Do you ever speak to friends or family about your health?

Whose advice do you value most?

Are there others whose advice you also value?

Do you worry about your health?

Where do you get most of your medical information?

- What sources of medical information do you trust? Probe: TV, newspaper, magazines, friends, family, etc.

What do you think is the greatest health concern facing Canadians? Why?

What disease do you think claims the most lives amongst women in Canada? Why?

Men? Why?

What do you think is the greatest health problem facing South Asians in Canada? Why?

Do you think this health problem is different for South Asians in your country of origin? Why?

What do you think is the leading cause of death amongst South Asians in Canada? Why?

Are South Asians at higher risk of diabetes? Do you know any risk factors for diabetes?
