# Supplementary material for: A qualitative investigation of optimal perinatal health: the perspectives of south Asian grandmothers living in southern Ontario, Canada
Source: BMC Pregnancy Childbirth. 2020 Feb 17;20:113. doi: 10.1186/s12884-020-2762-0 (PMC7026998; doi:10.1186/s12884-020-2762-0)
Supplement: Supplementary file 3 — Additional file 2. (Supporting raw data) This file contains additional supporting raw data from each theme. [file 12884_2020_2762_MOESM3_ESM.docx]

**Pre-Conception Period**

This period is characterized mainly by “Habit Building”, with the three subcategories of:

1. Mental Health
2. Diet
3. Exercise

The pre-conception period involves building good, healthy habits. This includes learning to reduce stress and occupying oneself in positive mental and physical state. Keeping one’s mind at peace is considered an important step in preparing to bring a baby into the world. In addition to practices such as yoga, reading, and maintaining positive, happy relationships with friends and family, this is achieved first and foremost through a healthy, balanced diet, rich in vitamins and nutrition. Many grandmothers emphasize the importance of moderation in one’s diet, implying that a healthy diet is one characterized by balance, instead of specific or extreme food restrictions. Even when asked directly “what particular foods a mother should consume pre-conception”, most grandmothers answered quite generally, indicating that a “nutritious” or “proper” diet, full of vegetables, fruit, milk, lentils, and other whole grains is of the utmost importance.

In addition to maintaining a healthy diet, many grandmothers recommend that expecting mothers engage in light exercise, the most commonly quoted being walking and housework. Few grandmothers mentioned intentional, strenuous exercises such as weight bearing, running, or gym-related activities, and instead focused on the importance of staying moderately active. Some grandmothers also express concern regarding more rigorous activities, as they believe them to adversely affect the expecting mother’s health by increasing the risks of abortion.

**Quotes**

1. Mental Health

- “Your mind should be healthy. You have to keep your mind peaceful.”
- “She has to stay happy and healthy. Most thing is happy, no stress.”
- “Well actually the most important thing I feel in any generation for that matter is that to be parents, both of them have to be mentally and financially ready to have a child.”

1. Diet

- “I think exercise is good. Eating healthy is good. I believe in moderation in everything you know. I don’t believe in extremes.”
- “Should be a well-balanced diet, not something in particular.”
- “I don’t think there’s anything they should not [eat], if they eat everything in moderation they should be fine.”

1. Exercise

- “Yeah they can do moderate exercise until the baby’s born… Walking, walking is the most they recommend.”
- “Walking. I wouldn’t do anything too strenuous to excess you know, no jogging or anything just walking on a treadmill or something.”
- “She should start with regular housework then gradually go for walks.”
- “Nothing too rigorous, she doesn’t want to abort.”

**Pregnancy Period**

This period is characterized by the maintenance of an “Enriched Environment”, which can be achieved by continuing to practice the healthy habits of the pre-conception stage.

Subcategories/ Themes Include:

1. Imprinting
2. Positive Relationships/ Support Systems
3. Routine/ Maintaining a Sense of Normalcy
4. Nutritional Enhancement
5. Exercise

The pregnancy period primarily involves maintaining an enriched environment, which follows from the preservation of healthy habits. In addition to keeping one’s mind at peace, this period includes surrounding oneself with religious and intellectual stimuli like books and pictures, and also engaging in prayers and ceremonies. These activities are seen as important not only for the expecting mother’s own mental health, but for the well-being of the child. This belief is founded on the notion of “imprinting”, in which the maternal behaviours are seen as influencing the growing child’s health, temperament, and religiosity. Many grandmothers spoke of the mother-child connection that exists, and the ways in which the mother’s own health and state of mind can influence the child in-utero. For this reason, maintaining a positive state of mind was seen as imperative to the health of the child, and many grandmothers spoke of different ways for the expecting mother to remain happy and stress-free. This often included being social, interacting with friends and family, and maintaining a sense of “normal” life and routine. Some grandmothers spoke of their own experience dealing with the stresses of pregnancy, and the importance of reaching out to support networks and asking for help when necessary.

Furthermore, the pregnancy period necessarily includes paying attention to one’s physical health, which can be maintained through a healthy diet and moderate activity. Foods are generally seen as nourishing as opposed to something to avoid, and there is an emphasis on nutritional enhancement in regards to adding special foods to the diet and replacing/ replenishing vitamins. This also involves the idea that particular foods foster particular traits in the child and inform his or her overall health and well-being. Smoking, drinking, and the use of drugs is generally seen as something negative, both culturally and also because of the adverse health effects these substances can have. This viewpoint is generally extended to prescription medications, unless it is a last resort. Consistent with the pre-conception period, light exercise is seen as important, especially in managing pregnancy weight gains. While food is seen as nourishing and healthy, there is still an understanding that the pregnancy period should not be one of gluttony and inactivity, and the mother must still remain healthy and active for herself and the baby.

**Quotes**

1. Enriched Environment

- “Read good books, religious books… Read good novels and watch religious movies, or do prayer. Meditation.”
- “You can read books, visit spiritual institutions, listen to songs, radio, read newspapers, and talk to the public/ people.”

1. Imprinting

- “After the first three months I think the baby starts to hear what is going on in the family so it’s good to have good thoughts, read good books and listen to good music, and no fighting, you know the baby can tell when the mother’s upset. So psychologically try to have happy thoughts.”
- “I’ll have to go back on my own experience which I passed on to my daughter. Taking care of herself prior to giving birth you know, doing all that massaging stuff you know physical stuff. Mentally soothe yourself you know, listen to calming music. You know all of that is beneficially to the baby and I guess it’s all tied in to the same theme. The mother is calm the baby is going to be calm. The mother eats right the child will eat right so it’s all like a tree giving forth good food.”
- “Babies are not going to be born healthy. [For] a healthy baby, mom has to be healthy.”

1. Positive Relationships/ Support Systems

- “I think she has to be in a loving relationship with her spouse. I think that is key… You need that support system.
- “Call her mother, call her sister-in-law, call somebody and say please come over. You know don’t hesitate to call for help.”
- “She should talk to her husband, mother, and friends [if she felt sad during pregnancy]. You could go to meetings or to the temple. She can read books, watch television shows and remain happy.”

1. Routine/ Maintaining Sense of Normalcy

- “Yes, a pregnant woman can continue to work (if her health allows), as it will keep her fit. She can leave the work two weeks before she is ready to deliver.”
- You should carry on as normal. And don’t be depressed.”

1. Nutritional Enhancement

- “I think that’s an important thing, diet is important; you watch your diet you have a good baby.”
- “Well firstly I don’t agree with a mother who drinks alcohol, smokes, drinks first of all maybe give up caffeine and all that. Drink plenty of milk, fresh fruit. A healthy diet is most important.”
- “A pregnant woman should not eat what she cannot digest. She should eat nutritious food, which is good for her as well as for the baby.”
- “She should take care of herself as well as her child. She should eat healthy food, fruits and drink milk. If you eat well, your health will remain good and the child is bound to grow well.”

1. Exercise

- “She should work, walk and remain active.”
- “She should not gain too much of weight. Staying healthy and active is important.”
- “She should exercise and do household work. Sleeping entire day is not good, walking is a good exercise.”
- “She shouldn’t become obese for sure. You know that the baby only weighs on average 7 lbs and the placenta and fluid around it may be another… 20 lbs she can gain.”

**Post-Partum Period**

This period is characterized by “Healing and Restoration”, with subcategories including:

1. Self-Care
2. Positive Relationships/ Support Systems
3. Re-Building Healthy Habits
4. Mother-Child Connection
5. Healing Touch
6. Warmth and Calming Heat

The post-natal period is a time of healing and restoration for the mother and the baby. First and foremost, many grandmothers emphasized the importance of self-care during this period, ensuring that the mother regains both the physical and mental strength necessary to take care of the baby. This includes getting adequate rest, eating well, taking soothing baths, listening to calming music, and maintaining a positive mental state. Friends and family can play important role during this period by offering their love and support for the mother and new baby. Raising the child is often seen as a collective responsibility, and many grandmothers assume an active role in this regard. Furthermore, this ongoing practical and emotional support is seen as imperative to the mother’s mental health, helping her during this speriod of stress and change. Following this initial period of recovery and restoration, the mother is encouraged to start re-building healthy habits and re-integrating into the flow of normal life. This often includes becoming physically active, eating healthy, and maintaining a good social network.

Furthermore, during the period, the mother also begins to bond with the child through co-sleeping, massages/ stretching, breast feeding etc. Breastfeeding is seen as important and often linked to reduced infection and sickness in the baby. Similar to the notion of imprinting, there is a belief that the baby can sense when the mother is anxious or sad, and therefore the mother’s mental state can have a profound affect on the child. Because of this “mother-child connection” it is imperative to avoid any stress or fighting that could influence the child’s temperament or impede the mother’s ability to take good care of the child. Furthermore, it is important to keep the baby warm and clean and surrounded by positive environments. As opposed to food being almost exclusively seen as nourishing during pregnancy, there was a focus on avoiding certain foods that would cause distress in the baby through breastfeeding (such as Kirandhi foods), as well as ensuring adequate nutritional enhancement.

**Quotes**

1. Self-Care

- “She should get as much rest as possible… she has to gain her own health first in order to be able to look after her own little one.”
- “She should look into her own self first and see what would give her, what used to make her happy, what used to give her more satisfaction… Maybe read, hear good music, go out… Essentially you should love your own self and your own company. If you can manage to get that down you will never be sad.”
- “I think a hot bath before bed works. Making sure, taking care of herself you know. Your body is not the same anymore and I found that after the entire day a hot bath suits.”
- “During the first month, the woman has to take proper care and rest to get normal.”

1. Positive Relationships/ Support Systems

- “If she’s anxious she should talk to her pediatrician or her doctor. Then she should talk to her friend who may have more experience, and then she should talk to her mother or aunt or people that have gone through it and they can give her comfort that you know this will pass, you’ll get your sleep you will be able to go back to work. She needs reassurance. And her health. She should get post-partum depression. That’s a very common thing.”
- “I think that the mother needs somebody there every day until she’s six weeks old. My daughter is still taking antidepressants til now, from the first baby… I don’t know how much care she had because you can’t interfere with certain things, and I don’t think she had the support like we had back home in our country, and I think she suffered because of that.”

1. Re-Building Healthy Habits

- “In the first year she should gradually get back into her own routine life. Because the longer a young new mother delays getting back into routine life.”
- “Nobody wants to go to work but in order to have a better life and a sense of individuality, independence – she should keep up her life, why should she change that?”
- “Not stay home a lot, go out, have a normal life. Do as best you can.”
- “She should be back to her routine.”

1. Mother-Child Connection

- “For one thing, it helps the baby have a healthy growth. Secondly it sort of builds a bond with the mother and the child. I know there’s a natural bond that comes with the mother and the child and a child gets more immunity. A child is healthier.” (Regarding breast-feeding)
- “A mother should be relaxed herself first because a child can immediately sense if his or her mother is tense about something. If his or her mother is sad, you may be surprised how these little ones can sense that especially if they are in the mother’s arms.”
- “Spend as much quality time with the baby, bond with the baby. Take time for herself. Rest. Eat healthy.”
- “Try and rest. Because I think if the mother is upset or anxious in any way I think the baby picks up on those vibes. So if the mother isn’t calm the baby’s not going to be calm.”

1. Healing Touch

- “You cross their arms, you cross their legs, you have their legs touching their head you know so it’s a stretching thing and the baby’s so relaxed they go to sleep.”
- “Even now my daughter-in-law massages the back and the tummy, soothes, reads to them, lullabies.”

1. Warmth and Calming Heat

- “Well I think the main thing is to make sure the baby’s warm. So it should have you know in this tiny little crib, pillows around so that the baby doesn’t feel cold because if it is cold it will cry. So keep the baby warm, well tucked in.”
- “The baby needs care and love. If you give them your warmth, they will sleep.”
- “A newborn should sleep with the mother as he/ she requires [the] warmth of the mother.”

**Advice**

As this study revolves around the advice that grandmothers give to expecting mothers, I thought it would be interesting to note the factors that inform their beliefs, and the ways in which they deliver their advice. I’ve identified three main sources of knowledge: experiential, medical-based, and community-based/ cultural. Throughout the interviews, the grandmothers referred back to all three sources, with varying attitudes towards them. Although it varied from interview to interview, there seemed to be a general consensus that the best advice was derived from one’s own experience, with additional support from a family physician or medical authority. Culturally specific sources of knowledge were still valued, but not necessarily above experience and Western medical advice. In general, the grandmothers appeared flexible in their beliefs, and seemed to impart the advice that they felt would best benefit the child.

**Quotes**

- “Even for infection, you have to take the baby to the doctor. You cannot cure it yourself.”
- “Well I suppose advice from an experienced friend, from an experienced mother, advice from maybe your doctor, if the child has a medical problem.”
- “Well you have to make a compromise. The friend is giving you advice from experience, and the doctor is giving you advice from knowledge or the medical side, so both are valuable actually.”
- “First to the doctor, then they will recommend her.”
- “Sometimes friends and family because they see other doctors and they give good advice that their doctor gave and you can put together all the advice.”
- “I would say first of all my family doctor. If I am experiencing something I’ll go and see him and we take a very conservative approach. But I do discuss it with friends and listen to what they have to tell me and maybe entertain the idea a little bit.”
- “I learnt it through my own experience.”
- “A lot of the advice I got I found was good advice, sound advice, but things are so different now. Like as I said my daughter-in-law and my daughter, they have 20, 30, 50 books about children from infancy to so many months and so many months and I’m looking at them and saying “Oh my god, they kids grew up fine.”

Attitudes towards giving advice can be categorized as follows:

1. Enforces: When the grandmother is steadfast in giving advice and believes that advice will be heeded. Stems from perceived role of the grandmother as a figure of wisdom and experience, one who is responsible for taking care of the family through her actions and the advice she provides.
2. Imparts [when asked]: When the grandmother imparts advice when requested, but does not necessarily advise otherwise. Stems from a belief or understanding that the daughter or daughter in law will act in the child’s best interest. Does not feel the need to enforce advice because the grandmother trusts the new mother and acknowledges generational and intercultural differences in terms of child-rearing.
3. Intervenes [when necessary]: When the grandmother only intervenes with advice when they see fit.

**Quotes**

1. **Enforces (need to find more quotes for this)**

- “Yes, of course.” – When asked “Do you advise her on childcare and healthcare?”
- “Yes, they listen. If they seek my suggestion they listen and follow as well.”
- “I also provide guidance to my daughter as to what she must do… Advice to sing well, read good books, practice healthy things… remembering and doing intelligent things.”
- “Extremely involved [in the family]… Yes, that would be the thing, respects my advice.”
- “Yes, they do listen. I suggest them about household matters.”
- “We do teach them a lot. We advise them on each matter and they do follow them.”

1. **Imparts**

- “I normally don’t give advice because I don’t like to impose myself… But yes whenever she does need any advice I do want to share my advice and she follows it. At other times I just give a suggestion for her to weigh the pros and cons and then decide for herself.”
- “She views me as a, I don’t know hopefully a good mother-in-law but she kind of, not looks up to me but she’s always asking for my advice, I teach her of our culture, cooking, everything.”
- “Well I realize that they are doing the right thing and for me a mother always feels that her daughters, no matter how old they are, need advice.”
- “A lot of the advice I got I found was good advice, sound advice, but things are so different now. Like as I said my daughter-in-law and my daughter, they have 20, 30, 50 books about children from infancy to so many months and so many months and I’m looking at them and saying “Oh my god, they kids grew up fine.”
- “These days as I said these kids are so well educated they have hundreds of books to read from. They go online and check everything so as far as giving them advice I mean there’s not much I can tell her not to do.”
- “I’ve always found my daughter-in-law that she is well, she knows what she has to do and not do.”
- “If they come to us, we teach them.”

1. **Intervenes**

- “Or when she would feel very tired… That’s when I would point out to her yes this is what’s lacking and this is what’s exceeding.”
- “My role is redundant now, because I can’t give too much advice because… my advice is usually about the children.”
- “I meant that should be her choice as to what she wants to eat or drink, but as to what you want to advocate it’s different… Like iron… take supplements if she’s low on iron.”
- “Because I’m very good at researching diet in any condition… I talk to them during, before the pregnancy and after, and even though the kids are grown up… with their diet [you] [have] to make sure they eat the food the body needs and not what the mouth desires.”
- “I wouldn’t go with too much of sterilized atmosphere because the body needs to start to develop you know, some sort of immunity. Cause I see my son doing that a lot right now… I said you are harming them because they need to be exposed to some things.” – in response to the question: “Are there any consequences to keeping the baby too clean or too dirty?”

**Role of the Grandmother**

The grandmother generally assumes a very gender-specific role that includes cooking, cleaning, and taking care of the family. The grandmother plays an important role in the familial hierarchy and is seen [or sees herself] as a figure of wisdom and experience, often advising their daughters and daughters-in-law on matters regarding food, activity, and optimal maternal behaviours. There is also the idea that the grandmother is the one who preserves the culture and traditions from back home. Furthermore, the grandmothers often maintain a very positive relationship with their grandchildren, and enjoy taking an active role in their health and well-being. This relationship is often associated with a great sense of fulfillment and purpose.

**Quotes**

- “Oh, cooking, cleaning, take care of the children – that is my role.”
- “I love to handle children. I am always interested in my grandchildren’s growth, studies and health. It’s my choice too. I love to take care of them.”
- “We keep our culture and religion.”
- “I’m the caregiver of the family. I have to watch everybody [and know] what they’re doing.”
- “We have such an active role in their upbringing so it’s great.”

**Health Literacy**

At the end of each interview, the grandmothers were asked a few questions to gage their health literacy levels, and they were also asked specific questions regarding the biggest health concerns in their communities. Most grandmothers acknowledged the propensity of South Asians in Canada to be at risk of heart disease and diabetes, and when asked for probable causes, many cited both lack of exercise and poor diet.

**Quotes**

- “Heart disease and diabetes. When they migrate and come into Canada they see a lot of foods that are so attractive that they may not have had back home so they tend to have a lot of that and over intake of the wrong type of foods gets them into these health issues.”
- “I think it’s dietary mainly, you know too much carbohydrates, sugary desserts, lack of exercise and stress of coming to a new country.”
- “I think it’s the same thing they say diet causes, sedentary lifestyle you know sit there have the belly keep growing…. You know not choosing to have a home cooked meal is a big mistake. Simple.”
- “Uh, it is slightly different because our winters are severe so we’re used to working those 7,8 hours, you come home, you watch TV, you eat dinner and there’s no going out for a walk or anything because of the severity of the weather. Whereas in India I think people are still pretty you know active. And I don’t think they indulge in food like we do here.”
